# Implication of inflammatory signaling pathways in obesity-induced insulin resistance

**DOI:** 10.3389/fendo.2012.00181

**Published:** 2013-01-08

**Authors:** Jean-François Tanti, Franck Ceppo, Jennifer Jager, Flavien Berthou

**Affiliations:** ^1^INSERM U1065, Mediterranean Center of Molecular Medicine (C3M), Team “Molecular and Cellular Physiopathology of Obesity and Diabetes”Nice, France; ^2^Faculty of Medicine, University of Nice Sophia-AntipolisNice, France

**Keywords:** obesity, insulin resistance, inflammation, adipose tissue, pattern-recognition receptors, stress kinases, macrophages

## Abstract

Obesity is characterized by the development of a low-grade chronic inflammatory state in different metabolic tissues including adipose tissue and liver. This inflammation develops in response to an excess of nutrient flux and is now recognized as an important link between obesity and insulin resistance. Several dietary factors like saturated fatty acids and glucose as well as changes in gut microbiota have been proposed as triggers of this metabolic inflammation through the activation of pattern-recognition receptors (PRRs), including Toll-like receptors (TLR), inflammasome, and nucleotide oligomerization domain (NOD). The consequences are the production of pro-inflammatory cytokines and the recruitment of immune cells such as macrophages and T lymphocytes in metabolic tissues. Inflammatory cytokines activate several kinases like IKKβ, mTOR/S6 kinase, and MAP kinases as well as SOCS proteins that interfere with insulin signaling and action in adipocytes and hepatocytes. In this review, we summarize recent studies demonstrating that PRRs and stress kinases are important integrators of metabolic and inflammatory stress signals in metabolic tissues leading to peripheral and central insulin resistance and metabolic dysfunction. We discuss recent data obtained with genetically modified mice and pharmacological approaches suggesting that these inflammatory pathways are potential novel pharmacological targets for the management of obesity-associated insulin resistance.

## Introduction

Obesity is characterized by an excessive adipose tissue expansion due to an increase in nutrients intake and insufficient energetic expenditure. Obesity has dramatically increased worldwide and leads to numerous adverse metabolic disorders including cardiovascular diseases, type 2 diabetes, and some forms of cancer. Insulin resistance is associated with obesity and is a central component of type 2 diabetes, leading to altered glucose and lipid metabolism in adipose tissue, liver, and skeletal muscles. Insulin resistance is characterized by a decrease in insulin signaling mainly in the Insulin Receptor Substrate (IRS)/PI-3-kinase/PKB axis that is responsible for most of the metabolic actions of the hormone (Taniguchi et al., 2006). It is now recognized that a chronic low-grade systemic and local inflammation that develops during obesity could connect obesity to the development of insulin resistance (Gregor and Hotamisligil, 2011). This inflammatory state has been reported in different organs involved in the control of metabolic homeostasis including adipose tissue, liver, endocrine pancreas, hypothalamus, and possibly skeletal muscles. The chronic inflammation is caused by an excess of nutrient intake and has been named metabolic inflammation or metainflammation (Gregor and Hotamisligil, 2011). Several dietary factors including saturated fatty acids and glucose as well as a change in gut microbiota have been proposed as triggers of this metabolic inflammation that involves both metabolic cells, such as adipocytes, and a change in the population of immune cells in metabolic tissues (Lolmede et al., 2011; Bertola et al., 2012; Sun et al., 2012). Hypoxia that develops in adipose tissue could also participate in its inflammation and has been recently involved in insulin resistance of adipocytes (Regazzetti et al., 2009; Wood et al., 2009).

In this review, we will first describe the major mediators that link the excess of nutrients to the production of inflammatory cytokines, focusing on pattern-recognition receptors (PRRs). We will then discuss the intracellular signaling pathways activated by inflammatory mediators and involved in the desensitization of insulin signaling. We will also discuss whether and how the blockade of these mechanisms could improve insulin sensitivity.

## Immune sensors linking nutritional stress to obesity-induced inflammation and insulin resistance

### Toll-like receptors

Toll-like receptors (TLR) belong to the family of PRRs and play a crucial role in innate immunity by their ability to sense pathogens through the pathogen-associated molecular patterns (PAMPs) and to detect tissue injury through the danger-associated molecular patterns (DAMPs) (Mogensen, 2009). TLRs 1/2/4/5/6/11 are plasma membrane proteins whereas TLRs 3/7/8/9 are present in intracellular compartments. Microbial components induce the activation of the TLR signaling through a MyD88 (myeloid differentiation factor)-dependent pathway, except for TLR3, leading to the activation of the transcription factors NF-κB and AP-1 and the production of inflammatory cytokines. Mitogen-activated protein kinases including extracellular signal-regulated kinases (ERK1/2), JNK, and p38 are also activated by TLRs engagement. TLR3 and TLR4 activation also induces an IFN-β response through a MyD88-independent but TRIF (TIR domain-containing adaptor inducing interferon)-dependent pathway (Mogensen, 2009).

**Figure 1:**
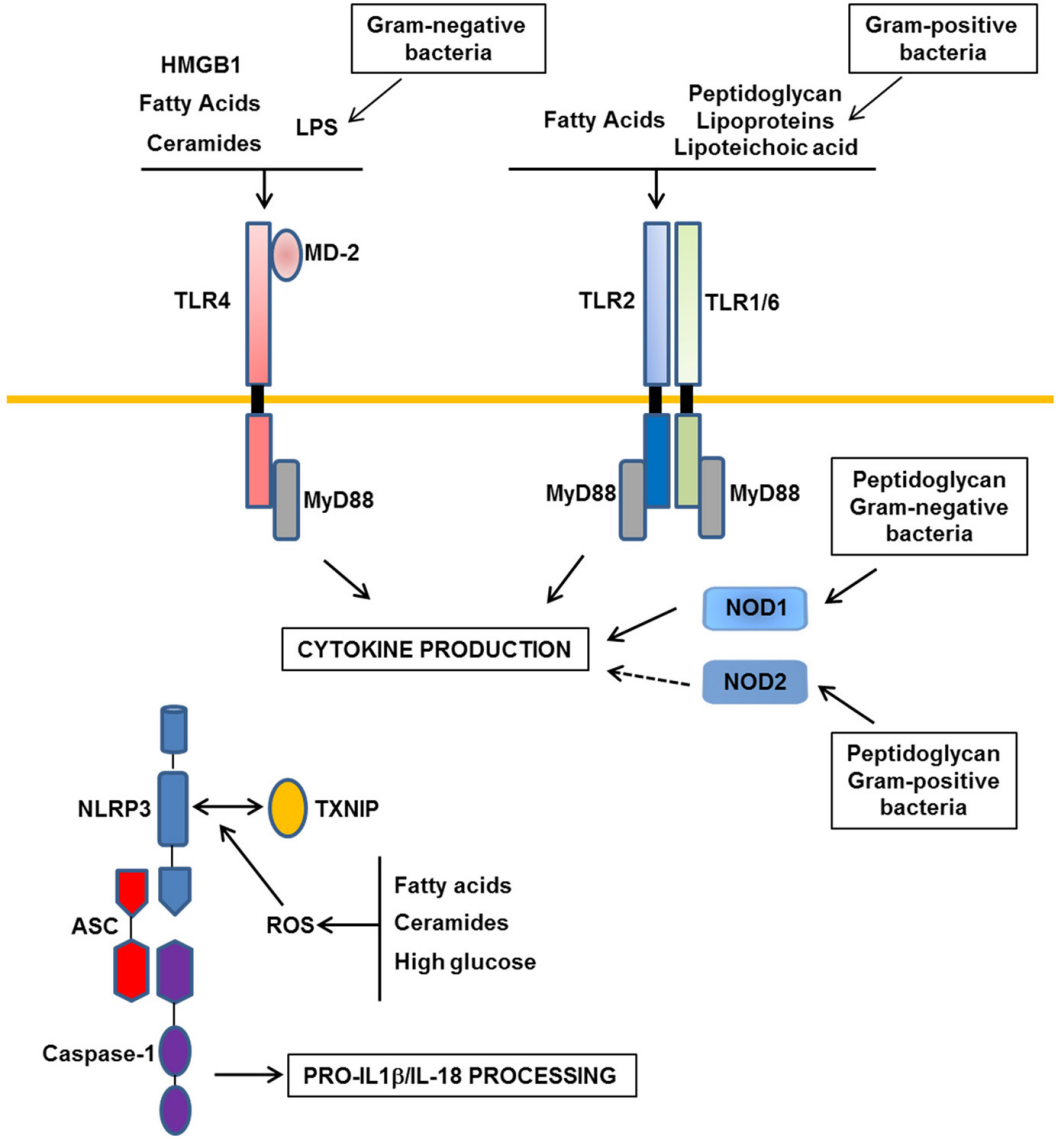
**Major Patterns-Recognition Receptors involved in obesity-induced inflammation.** In obesity, the moderate increase in LPS derived from gram-negative commensal bacteria activates TLR4 (Toll-like receptors). TLR2 and NOD1/2 (Nucleotide Oligomerization Domain) could be activated by peptidoglycan, lipoproteins, and lipoteichoic acid from gram-negative or -positive commensal bacteria. In addition, nutrients such as saturated fatty acids and their metabolites ceramides could interact with TLR4 or could indirectly activate this receptor through the production of DAMPs such as HMGB1. TLR2 could also be a receptor for saturated fatty acids. Following activation of these receptors, inflammatory cytokines are produced. Among them, IL-1β and IL-18 should be processed in their mature forms by the NLRP3 inflammasome composed of NLRP3, ASC, and caspase-1. ROS production in response to a high level of fatty acids, ceramides, or glucose could trigger the association between TXNIP and NLRP3 leading to the activation of caspase-1 in the inflammasome complex.

Among the different members of the TLR family, several groups have reported a role for TLR2, TLR4 in inflammation and insulin resistance during obesity (Fresno et al., 2011; Könner and Brüning, 2011). Most of the studies focused on TLR4 which is expressed in macrophages, dendritic cells but also in adipocytes, hepatocytes, muscles, and in the hypothalamus. TLR4 expression is increased in obese mice and obese and diabetic patients and negatively correlates with insulin sensitivity (Könner and Brüning, 2011). Recently it has been proposed that during obesity, metabolic endotoxemia contributes to the development of inflammation and metabolic disorders through the activation of TLR4 in metabolic tissues (Figure 1). Metabolic endotoxemia is defined as a moderate increase in circulating lipopolysaccharide (LPS) from Gram-negative bacteria and it develops owing to alterations in the composition of gut microbiota and to an increase in gut permeability (Cani and Delzenne, 2009; Burcelin et al., 2011). Further, high-fat diet could also enhance the translocation of live Gram-negative bacteria from the gut to the adipose tissue, a process that is dependent, at least partly, on CD14 that acts as a co-receptor with TLR4 to sense LPS (Amar et al., 2011).

Saturated fatty acids are other potential ligands of TLR4 both in adipocytes and macrophages leading to the production of inflammatory cytokines and also ceramides (Shi et al., 2006b; Radin et al., 2008; Fresno et al., 2011; Könner and Brüning, 2011) (Figure 1). TLR4 also mediates the cross-talk between adipocytes and macrophages induced by fatty acids (Suganami et al., 2007b). However, a direct binding of saturated fatty acids to TLR4 has been recently challenged due to LPS contamination (Erridge and Samani, 2009). It is thus possible that saturated fatty acids indirectly interact with TLR4 through fetuin A (Pal et al., 2012). Alternatively, fatty acids might activate TLR4 signaling through the production of endogenous DAMPs such as HMGB1 (High-Mobility Group Box1) and/or through ceramides production (Fischer et al., 2007; Li et al., 2011) (Figure 1).

The *in vivo* pathophysiological importance of TLR4 in obesity-induced inflammation and insulin resistance was investigated by using mice deficient in TLR4 signaling owing to invalidation of TLR4 (TLR4^−/−^ mice) or to a loss-of-function mutation in the *Tlr4* gene (C3H/HeJ and C57BL/10ScN) (Table 1). The different studies have reported a mild reduction in inflammation in adipose tissue and liver or in the vasculature (Shi et al., 2006b; Kim et al., 2007; Poggi et al., 2007; Suganami et al., 2007a; Tsukumo et al., 2007; Li et al., 2011; Orr et al., 2012; Ye et al., 2012). The lower inflammation in adipose tissue was linked to a decrease in macrophage infiltration or to a change in macrophage polarization toward a M2 anti-inflammatory phenotype (Shi et al., 2006b; Tsukumo et al., 2007; Davis et al., 2008; Orr et al., 2012). Reduction in inflammation in liver Kupffer cells and in liver parenchymal cells (Li et al., 2011; Ye et al., 2012) was associated with a decrease in hepatic steatosis or with a reduction in the progression from steatosis to non-alcoholic steatohepatitis (Poggi et al., 2007; Tsukumo et al., 2007; Radin et al., 2008; Li et al., 2011; Orr et al., 2012; Ye et al., 2012). In contrast, contradictory results have been obtained concerning the development of obesity. Some studies have reported that C3H/HeJ, 10ScN, or male TLR4^−/−^ mice gained less weight on a high-fat diet than their respective controls (Tsukumo et al., 2007; Davis et al., 2008; Radin et al., 2008; Saberi et al., 2009; Orr et al., 2012). This phenotype could be related to a protection against diet-induced leptin or insulin resistance in the hypothalamus in the absence of a functional TLR4 signaling (Kleinridders et al., 2009; Milanski et al., 2009; Könner and Brüning, 2011). However, other studies have described a higher feeding efficiency of the C3H/HeJ mice with increased adipose tissue mass and adipocyte hypertrophy (Poggi et al., 2007), an increase in body weight gain and adipose tissue mass in female TLR4^−/−^ mice (Shi et al., 2006b) or no protection against obesity in male TLR4^−/−^ mice (Shi et al., 2006b; Kim et al., 2007). The majority of the studies have reported a reduction in the insulin resistance, at least in adipose tissue and liver (Shi et al., 2006b; Suganami et al., 2007a; Tsukumo et al., 2007; Poggi et al., 2007; Davis et al., 2008). However, some of them, mainly concerning male TLR4^−/−^, did not show any improvement in whole-body insulin sensitivity (Shi et al., 2006b; Radin et al., 2008; Ding et al., 2012; Orr et al., 2012).

**Table 1:** **Phenotype of the different TLR4-deficient mice fed with a high-fat diet**.

	**Genotype**	**% Fat in diet**	**Body Weight gain**	**Whole-Body IS**	**Food Intake**	**Adipose Tissue inflammation**	**ATM**	**Adipose Tissue IS**	**Steatosis**
Tsukumo et al.	C3H/HeJ	55	↓	↑	=	↓	↓	↑	↓
Poggi et al.	C3H/HeJ	45	=	=	↓	↓	=	↑	↓
Suganami et al.	C3H/HeJ	60	=	↑	ND	↓	=	ND	ND
Davis et al.	10 ScN	60	↓	↑	ND	↓ ±	↓	ND	ND
Radin et al.	10 ScN	45	↓	=	↓	ND	ND	ND	↓
Li et al.	10 ScN	60	↓		=	ND	ND	ND	↓
Shi et al.	TLR4^−/−^ F	60	↑	↑	↑	↓	↓	ND	ND
Shi et al.	TLR4^−/−^ M	60	=	=	=	↓	ND	ND	ND
Orr et al.	TLR4^−/−^	45	↓	=	↓	↓ ±	↑ M2	ND	↓
Saberi et al.	TLR4^−/−^	No data	↓	ND	ND	ND	ND	ND	ND
Ding et al.	TLR4^−/−^LDLR^−/−^	35.5	=	=	=	=	=	ND	ND
Kim et al.	TLR4^−/−^	60	=	ND	ND	ND	ND	ND	ND
Saberi et al.	BMT-10ScN	No data	=	↑	=	↓	↓	↑	↓
Orr et al.	BMT-TLR4^−/−^	45	=	=	ND	↓	↑ M2	ND	=
Coenen et al.	BMT-TLR4^−/−^	41	=	=	ND	=	=	ND	ND

C3H/HeJ: Mice harboring a spontaneous missense mutation in the third exon in the Tlr4 gene leading to a loss-of-function of TLR4.

10ScN: Mice that display a spontaneous mutation resulting in 7 kb deletion in the Tlr4 gene that results in absence of both mRNA and protein expression.

TLR4^−/−^: Mice with a knockout of the Tlr4 gene. F female. M male.

BMT-10ScN: Transplantation of bone marrow from 10ScN mice into C57BL6 mice.

BMT-TLR4^−/−^: Transplantation of bone marrow from TLR4^−/−^ mice into C57BL/6 mice (Orr et al) or into agouty (A^y^/a)/LDL-receptor deficient mice (A^y^/a;Ldlr^−/−^) (Coenen et al.).

IS: Insulin Sensitivity.

ATM: Adipose tissue macrophages.

M2: macrophages with a M2 anti-inflammatory polarization.

ND: not determined in the study.

Different studies have investigated the consequences of TLR4 invalidation specifically in immune cells by transplantation of bone marrow from TLR4-deficient mice into wild-type recipients. In one study, mice developed obesity but with less inflammation and insulin resistance in adipose tissue and liver and no change in muscles (Saberi et al., 2009). In contrast, two other studies using the same experimental strategy but in different genetic backgrounds, failed to observe any improvement in insulin resistance despite a reduction in adipose tissue inflammation and/or a shift in adipose tissue macrophage polarization toward a M2 anti-inflammatory state (Coenen et al., 2009; Orr et al., 2012).

In summary, the consequences on weight gain and insulin resistance are different depending on the genetic background, the sex of the mice, the duration, and the lipid composition of the diet. In this regard, the percentage of saturated lipids in the diet could be important since the protection against insulin resistance seemed to occur selectively when the diet contained a high level of saturated lipids (Davis et al., 2008). It remains to be determined whether the activation of TLR4 signaling by a saturated fat diet is due to a direct action of fatty acids on TLR4 or to an indirect effect through the production of endogenous DAMPs or through the modification of the gut microbiota leading to an increase in circulating LPS. Further, clarification is needed for the respective contribution of TLR4 signaling in hematopoietic vs. non-hematopoietic compartment to the development of insulin resistance. Of note, the analysis of the metabolic phenotype of the TLR4-deficient mice could be complicated due to the potential compensatory increase in TLR2 expression (Ding et al., 2012).

TLR2 detects lipoproteins, lipoteichoic acid, and peptidoglycan from gram-positive bacteria and dimerizes with TLR1 and TLR6 (Mogensen, 2009). TLR2 has a broad pattern of expression and besides its expression in immune cells, is also expressed in insulin sensitive cells and islets (Ehses et al., 2010). A role for TLR2 in the metabolic complications of obesity was suggested by its increased level in different metabolic tissues of obese mice and patients and in circulating monocytes of type 2 diabetic patients (Fresno et al., 2011; Könner and Brüning, 2011). Like TLR4, TLR2 could be a sensor for saturated fatty acids mediating their pro-inflammatory effects in adipose tissue and macrophages and participating in the development of insulin resistance in cultured myotubes or adipocytes (Fresno et al., 2011; Könner and Brüning, 2011). However, as for TLR4, a direct activation of TLR2 by saturated fatty acids has been questioned owing to possible contamination by LPS (Erridge and Samani, 2009). Thus, both TLR4 and TLR2 could participate in the sensing of abnormal levels of nutrients, especially fatty acids and in the detection of gut microflora modification in obesity (Figure 1). Cooperation between these two TLRs might be involved since TLR4 activation increases the synthesis of TLR2 in adipocytes. This overlapping function could explain the similar metabolic phenotype of TLR4 and TLR2 deficient mice. Indeed, invalidation of TLR2 improved diet-induced insulin resistance and inflammation of adipose tissue, liver, or muscles (Caricilli et al., 2008; Kuo et al., 2011; Ehses et al., 2010 #3013, Himes and Smith, 2010 #3010). Further, TLR2 signaling is involved in diet-induced pancreatic islet inflammation and beta-cell dysfunction (Ehses et al., 2010). TLR2 knockout mice have also a reduced adiposity (Ehses et al., 2010; Himes and Smith, 2010; Kuo et al., 2011) suggesting that the lack of TLR2 signaling could decrease lipid uptake or increase lipid oxidation in different tissues. Whether the effect of TLR2 activation on lipid metabolism is direct or the consequence of an inflammatory state remains to be clarified. However, the reported interaction between activated TLR2 and CD36, a transporter of fatty acids, supports a role for TLR2 signaling in fatty acids uptake in metabolic tissues (Triantafilou et al., 2006).

Although the findings discussed above support a protective role of TLR2 inactivation in the context of obesity, a recent study reported that TLR2 knockout mice had a phenotype reminiscent of metabolic syndrome even on low-fat diet. In this setting, it was demonstrated that the gut microbiota was responsible for the development of insulin resistance (Caricilli et al., 2011). This finding illustrates the concept that complex interactions between environment, gut microbiota, and the genetic of the host drive the metabolic phenotype (Nicholson et al., 2012). In this regard, a specific environmental condition and the innate immune system may have shaped a harmful gut microbiota that overcomes the protective effect of the genetic deficiency in TLR2. Alternatively the loss of TLR in immune cells may alter gut microbiota leading to the development of inflammation, obesity, and insulin resistance as described for mice lacking TLR5 (Vijay-Kumar et al., 2010), a TLR highly expressed in the intestinal mucosa and involved in the detection of bacterial flagellin (Mogensen, 2009).

### Inflammasome and NOD

Nucleotide oligomerization domain (NOD) 1 and 2 are intracellular proteins that recognize cell wall peptidoglycan moieties from gram-negative or gram-positive bacteria, respectively (Mogensen, 2009). NOD proteins have recently emerged as immune sensors involved in inflammation-induced insulin resistance (Figure 1). Peptidoglycan-induced activation of NOD1 in adipocytes or hepatocytes (Schertzer et al., 2011; Zhao et al., 2011) and NOD2 in muscle cells (Tamrakar et al., 2010) trigger insulin resistance through the production of inflammatory mediators and the activation of MAP kinases signaling leading to desensitization of IRS1 function. Injection of specific NOD1 ligand in mice promoted adipose tissue inflammation and induced whole-body insulin resistance with a strong decrease in insulin action in the liver. NOD2 ligand injection caused a milder insulin resistance and preferentially in muscles (Schertzer et al., 2011). NOD1-deficient mice, but not NOD2, were protected against glucose intolerance and diabetes induced by a high-fat diet (Amar et al., 2011). These studies demonstrate the ability of NOD activation to induce insulin resistance and support the implication of NOD1 in the control of metabolic diseases through the sensing of components from gram-negative bacteria (Figure 1).

Inflammasomes are multi-protein complexes composed of three proteins: the nucleotide-binding domain leucine-rich repeat (NLR) protein, the adaptor protein ASC (apoptosis-associated speck-like protein containing a CARD) and the caspase-1. Four different inflammasomes have been identified so far namely NLRP1, NLRP3, NLRC4, and AIM2. Pathogen and danger-associated signals activate inflammasomes leading to the processing of IL-1β and IL-18 by caspase-1 (Mogensen, 2009). It is recognized that IL-1β is one of the main cytokines implicated in the desensitization of insulin signaling (Lagathu et al., 2006; Jager et al., 2007) and its genetic invalidation protects mice against diet-induced insulin resistance (Stienstra et al., 2010; Wen et al., 2011). Pharmacological inhibition of IL-1β signaling by the IL-1 receptor antagonist anakinra, mitigates inflammation, and improves glycemic control in type 2 diabetic patients (Larsen et al., 2007). With this in mind, several groups have investigated the implication of inflammasome activation in insulin resistance.

The expression of NLRP3 and caspase-1 is increased in adipose tissue of obese mice, overweight subjects, or obese individuals with type 2 diabetes (Stienstra et al., 2010; Koenen et al., 2011; Vandanmagsar et al., 2011). The identity of cells within the adipose tissue, in which the NLRP3 inflammasome is activated, remains controversial. Two studies reported an expression and activation mainly in adipose tissue macrophages with a low expression in adipocytes whereas Stienstra et al. found an important contribution of adipocytes (Stienstra et al., 2010; Vandanmagsar et al., 2011; Wen et al., 2011). However, invalidation of different components of the NLRP3 inflammasome (NLRP3, ASC, caspase-1) univocally protected the mice against high-fat diet-induced inflammation and insulin resistance. This phenotype was associated with a reduced expression of IL-1β in adipose tissue and a reduced level of circulating IL-18 (Stienstra et al., 2010; Vandanmagsar et al., 2011; Wen et al., 2011). The lack of the NLRP3 inflammasome had consequences on the subset of immune cells within the adipose tissue. The number of M2 anti-inflammatory macrophages was increased in subcutaneous adipose tissue (SAT) and the activation of pro-inflammatory macrophages was dampened in visceral adipose tissue (VAT). In parallel, the number of naive CD4^+^ and CD8^+^ T cells was increased in SAT and the amount of effector memory CD4^+^ and CD8^+^ T cells was decreased in VAT (Vandanmagsar et al., 2011). These findings suggest a model whereby the NLRP3 inflammasome-dependent production of IL-1β and IL-18 by adipose tissue macrophages favors macrophage-T cell activation leading to a sustained inflammation of adipose tissue (Vandanmagsar et al., 2011). Besides its role in the control of adipose tissue inflammation, inflammasome activation in adipocytes could limit energy expenditure, fat oxidation, and adipogenesis as revealed by the phenotype of the caspase1^−/−^ mice (Stienstra et al., 2010, 2011). In addition to adipose tissue, activation of NLRP3-inflammasome in other tissues such as pancreatic islets has been reported (Zhou et al., 2010).

These findings strongly support a model whereby danger signals generated in obesity are detected by the NLRP3-inflammasome that in turn promotes inflammation and the dysfunction of different organs involved in the control of glucose and lipid homeostasis (Figure 1). The identity of the danger signals that activate the NLRP3 inflammasome in obesity remains ill-defined. However, the observation that the NLRP3 inflammasome is activated by fatty acids and ceramides suggests that the lipotoxic environment in obesity might trigger its activation (Vandanmagsar et al., 2011; Wen et al., 2011). However, the NLRP3 inflammasome can be activated by other molecules such as ATP, glucose, oxidized LDL, uric acid, and crystals of cholesterol. Since all of them are elevated in obesity, their respective contributions to the activation of inflammasome deserve further investigation. The common feature of these danger signals is their ability to increase ROS production that is prerequisite for NLRP3 inflammasome activation (Tschopp and Schroder, 2010). Thus, the activation of NLRP3 inflammasome in obesity might be related to the oxidative stress that develops in the different metabolic tissues. How NLRP3 inflammasome is activated by ROS is not completely understood but the thioredoxin-interacting protein (TXNIP) has recently emerged as a potential link with the demonstration of its binding with NLRP3 in a ROS sensitive manner leading to NLRP3 inflammasome activation (Zhou et al., 2010) (Figure 1). This function might explain the similar phenotype between TXNIP- and NLRP3-deficient mice when fed a high-fat diet (Zhou et al., 2010). Further TXNIP might also connect organelle stress, such as reticulum endoplasmic stress, to NLRP3 inflammasome activation (Oslowski et al., 2012).

All together, these findings render the NLRP3 inflammasome as an attractive pharmacological target against the complications of obesity. In this multi-proteins complex, the easiest target is the caspase-1 since inhibitors already exist. The caspase-1 inhibitor Pralnacasan reduced body weight and improved insulin sensitivity of genetically obese *ob/ob* mice (Stienstra et al., 2010). However, one important caveat for the treatment of a chronic disease such as diabetes is that caspase-1 is involved in different inflammasome complexes and its inhibition may reduce the ability to fight infection.

Despite the potential role of the NLRP3 inflammasome in obesity-induced inflammation and insulin resistance, it remains to determine whether its activation is a primary event in the disease that drives the inflammation. Further, the relative contribution of NLRP3 inflammasome to the development of inflammation in the metabolic tissues and in the different subset of cells of these tissues should be clarified. The role of other inflammasome complexes also deserves investigation since ablation of NLRP3 markedly reduced but did not totally abrogate caspase-1 activation in adipose tissue or liver of obese mice (Vandanmagsar et al., 2011). Finally, other caspase-1 substrates besides IL-1β and IL-18 might be involved in the deleterious effect of inflammasome activation on metabolic control. In this regard, the transcription factors SREBPs are activated by caspase-1(Gurcel et al., 2006) and it was recently shown that SREBP-1a regulated the expression of inflammasome components in macrophages (Im et al., 2011). Thus, a feed-forward loop involving caspase-1 activation and SREBP might link lipid metabolism to the inflammasome activation in obesity.

In conclusion, it is now recognized that the immune sensors described above (TLR, NOD, inflammasome) and others such as the pathogen-sensing kinase (PKR) (Nakamura et al., 2010) participate in the development of the metabolic inflammation. As recently discussed by Gregor and Hotamisligil in an outstanding review (Gregor and Hotamisligil, 2011), it is possible that in obesity the levels of nutrient intake may rise enough to stimulate pathogen- and danger-sensing pathways ultimately leading to the activation of immune cells in the different metabolic tissues. In other words, the organism, in overfeeding situation, recognizes the nutrients as harmful biological molecules and activates pathways that are usually engaged by pathogen or endogenous danger signals. In addition, modification of the gut microbiota and of the intestinal permeability in obesity may fuel the organism with inflammatory molecules such as LPS and other bacterial antigens or favor the translocation of commensal bacteria within metabolic tissues (Cani and Delzenne, 2009; Amar et al., 2011; Nicholson et al., 2012). As a consequence, inflammatory cytokines are overproduced and they activate different signaling pathways in metabolic cells that desensitize insulin signaling and alter the expression of proteins involved in glucose transporter trafficking (Kaddai et al., 2009).

## Inflammatory signaling pathways involved in the desensitization of insulin action

### The SOCS proteins

**Figure 2:**
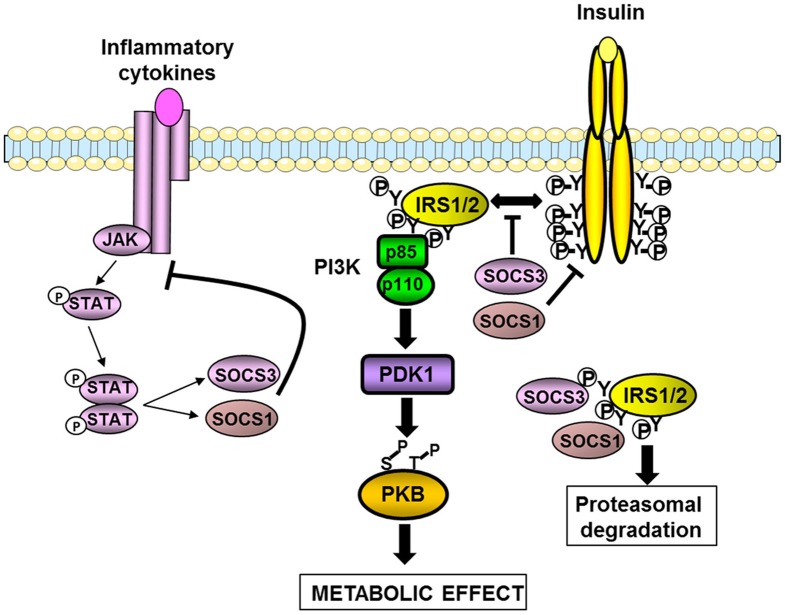
**Inhibition of insulin signaling pathway by SOCS1 and SOCS3.** SOCS1 and SOCS3 are induced by cytokines and involved in a negative feed-back loop. SOCS1 and SOCS3 also inhibit insulin signaling by different mechanisms. They interfere with the binding between the insulin receptor and IRS1/2 proteins. SOCS1 also inhibits the tyrosine kinase activity of the insulin receptor. Both SOCS1 and SOCS3 can interact with the tyrosine-phosphorylated IRS proteins leading to their degradation by the proteasome. The resulting effect is a decrease in the insulin-induced activation of the IRS1/2-PI3K-PKB axis leading to a reduction in the metabolic effects of insulin.

The Suppressor of cytokine signaling (SOCS) protein family also named Janus family kinase-binding (JAB) proteins or SSI (signal transducer and activator of transcription induced Stat inhibitor) includes eight members (SOCS1–7 and CIS), which possess a SH2 domain, and a SOCS-box domain controlling the degradation of interacting proteins. They are induced by several inflammatory cytokines and are involved in a negative feedback loop leading to the termination of cytokines action. At the molecular level, the SOCS proteins interact with the tyrosine kinases Janus-activated kinases (JAK) or directly with the receptor of some cytokines, thus blocking the tyrosine phosphorylation of the transcription factors STAT for review see Lebrun and Van Obberghen (2008). Several cellular studies have demonstrated that SOCS negatively regulate the signaling pathway of hormones including leptin and insulin. In this regard, SOCS3 is induced by leptin and insulin and is involved in a negative feedback loop and in a cross-down regulation (Emanuelli et al., 2000; Lebrun and Van Obberghen, 2008; Benomar et al., 2009). SOCS1, SOCS6, and SOCS7 are also involved in the desensitization of insulin signaling. SOCS3 inhibits insulin signaling by a direct binding through its SH2 domain with the juxtamembrane phosphotyrosine 960 on the insulin receptor, thus preventing the interaction of IRS1 and 2 with the receptor (Figure 2). SOCS1 interacts with the catalytic domain of the insulin receptor which contains an interaction motif for IRS2, blocking thus more selectively the tyrosine phosphorylation of IRS2. SOCS1 and SOCS6 also inhibit the tyrosine kinase activity of the insulin receptor. It has also been shown that SOCS proteins interact with the tyrosine phosphorylated IRS1 and IRS2 resulting in their ubiquitination and degradation by the proteasome (Lebrun and Van Obberghen, 2008) (Figure 2).

In obesity, inflammation leads to an up-regulation of SOCS proteins in hypothalamus, liver, muscles, and adipose tissue (Rieusset et al., 2004; Lebrun and Van Obberghen, 2008). A causal role for the up-regulation of SOCS proteins in the development of insulin resistance has been investigated in metabolic tissues. Overexpression of SOCS1 or SOCS3 in mouse liver or adipose tissue reduced the expression of IRS1 or IRS2 as well as their tyrosine phosphorylation induced by insulin. As a consequence insulin resistance developed in those tissues, as well as systemic insulin resistance for the overexpression in liver (Ueki et al., 2005; Shi et al., 2006a). Unexpectedly, overexpression of SOCS3 in adipose tissue protected the mice against systemic insulin resistance when fed a high-fat diet owing to a decrease in adipocyte hypertrophy (Shi et al., 2006a). Overexpression of SOCS3 in muscle exacerbated diet-induced obesity and insulin resistance but this effect was not due to a decreased insulin signaling but to an alteration in muscle integrity leading to a reduction in locomotor activity and energy expenditure (Lebrun et al., 2009).

The inhibition of SOCS proteins might thus be useful to prevent the development of obesity-induced insulin resistance. In agreement with such a possibility, heterozygous SOCS3 (SOCS3^+/−^) mice or mice with targeted invalidation of SOCS3 in the central nervous system (CNS) were protected against diet-induced obesity and associated insulin resistance (Howard et al., 2004; Mori et al., 2004; Kievit et al., 2006). This phenotype was explained by increased leptin sensitivity and possibly by an effect on insulin action for the SOCS3^+/−^ mice (Howard et al., 2004). In the same way, targeted invalidation of SOCS3 in adipose tissue or in muscles protected mice against obesity-induced insulin resistance (Jorgensen et al., 2012; Palanivel et al., 2012). SOCS7-deficient mice also displayed improved glucose tolerance and insulin sensitivity (Banks et al., 2005). However, several considerations should be taken into account before inhibiting SOCS proteins for therapeutic purpose. First, some SOCS proteins positively regulate insulin action as demonstrated by the improved insulin sensitivity of SOCS6-overexpressing mice (Li et al., 2004). Second, given that SOCS proteins negatively regulate the signaling of inflammatory cytokines, their chronic inhibition might exacerbate inflammation that could counterbalance its beneficial effect on insulin sensitivity. This dual action of SOCS proteins is well-illustrated by differential effects of short- and long-term invalidation of SOCS1 or SOCS3 in liver. Short-term invalidation by antisense oligonucleotides in obese and diabetic *db/db* mice improved hepatic steatosis with a mild reduction in insulin resistance (Ueki et al., 2005). In contrast, while targeted deletion of SOCS3 in liver-enhanced liver insulin sensitivity on chow diet, it accelerated the onset of high-fat diet- or age-induced insulin resistance with an increased inflammation in liver (Torisu et al., 2007; Sachithanandan et al., 2010). This dual action is not restricted to SOCS3, since SOCS1 in immune cells limited the metabolic inflammation in liver and perhaps adipose tissue (Sachithanandan et al., 2011). Thus, the beneficial effect of SOCS1-deletion on insulin sensitivity was only visible when SOCS1-deficient mice were crossed with interferon-gamma- or RAG2-deficient mice to limit inflammation. Of note, this beneficial effect was seen on chow diet (Jamieson et al., 2005; Emanuelli et al., 2008b) but was lost on a high-fat diet when the inflammation is enhanced (Emanuelli et al., 2008b). Further, SOCS2-deficient mice were protected against diet-induced hepatic steatosis probably due to an increase in the lipid mobilizing effect of growth hormone. However, this beneficial effect was overridden by a hyperproduction of inflammatory mediators by the resident macrophages leading to a higher level of inflammation in liver and adipose tissue and the worsening of insulin resistance (Zadjali et al., 2012).

In summary, it appears to date that SOCS3 is mainly involved in the regulation of energy balance through the down-regulation of leptin signaling whereas several SOCS such as SOCS1/3/6/7 are involved in the regulation of the insulin sensitivity. Since SOCS proteins contribute to the development of diet-induced obesity and insulin resistance, their targeting could be open new avenues for the treatment of metabolic disorders. However, careful examination of the balance between pro-inflammatory and insulin sensitizing effects of future inhibitors of SOCS proteins will be needed.

### The IKKβ/NF-κB and JNK pathways in inflammatory cytokines production and insulin resistance

**Figure 3:**
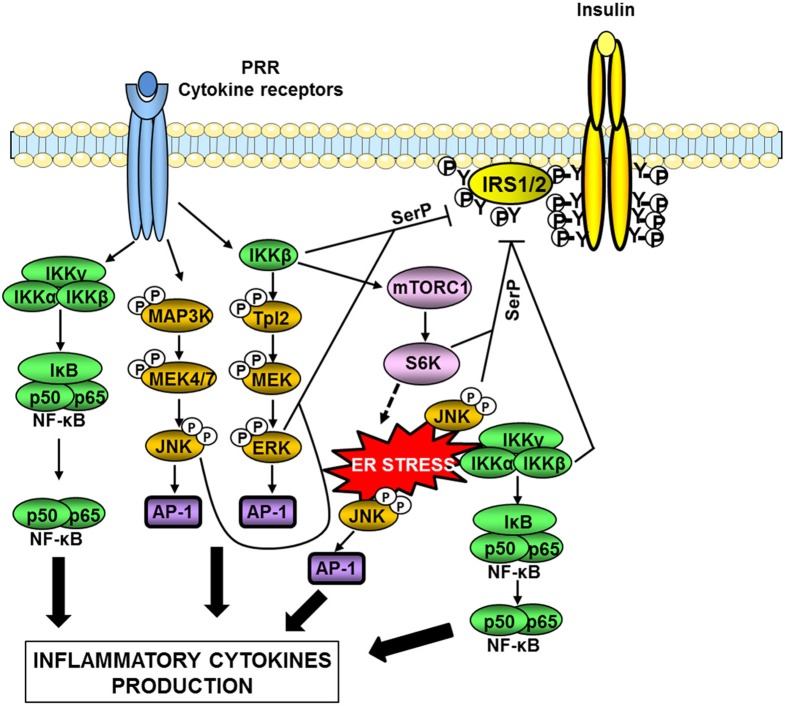
**Serine kinases involved in obesity-induced inflammation and insulin resistance.** In obesity, a network of serine kinases is activated including IKKβ, JNK, and ERK1/2. JNK and IKKβ are activated downstream of pattern-recognition receptors (PRRs) such as TLRs or by ER stress. IKKβ is also involved in the activation of the Tpl2/ERK pathway. These pathways participate in the production of inflammatory cytokines via the transcription factors AP-1 and NF-κB. Many of the produced inflammatory cytokines are able to activate these kinases leading to a feed-forward amplification loop. JNK and ERK1/2 are involved in the desensitization of insulin signaling through phosphorylation of IRS1/2 on inhibitory serine sites (SerP). IKKβ can directly phosphorylate IRS1/2 on serine sites but can also act indirectly through activation of mTORC1/S6 kinase. Over-activation of the mTORC1/S6K pathway could promote ER stress leading to an amplification loop.

The activity of both IκB-kinase β (IKKβ) and JNK is elevated in metabolic tissues in obesity, and these kinases are important nodes in the production of inflammatory mediators and in the desensitization of insulin signaling (Tanti and Jager, 2009; Solinas and Karin, 2010; Donath and Shoelson, 2011). JNK and IKKβ are activated downstream of immune sensors such as TLRs and participate in the production of inflammatory cytokines via the transcription factors AP-1 and NF-κB respectively (Figure 3). Many of the produced inflammatory cytokines are able to activate these two kinases leading to a feed-forward amplification loop (Donath and Shoelson, 2011; Gregor and Hotamisligil, 2011). Another important activator of these kinases is the endoplasmic reticulum (ER) stress (Figure 3).

ER stress occurs when the synthesis capacity of the ER is exceeded. In this case, the unfolded protein response (UPR) is activated in order to restore ER homeostasis. Three pathways are involved in the UPR, namely PERK (PKR-like eukaryotic initiation factor 2a), IRE-1 (inositol requiring enzyme 1), and ATF6 (activating transcription factor 6) pathways. If these mechanisms fail to restore proper ER homeostasis, cells undergo apoptosis (Xu et al., 2005). In a pioneer study, Hotamisligil and colleagues (Ozcan et al., 2004) have demonstrated that ER stress developed in liver and adipose tissue during obesity owing to nutrient overload and participated in the onset of insulin resistance. Several recent reviews have discussed in details the mechanisms linking ER stress to obesity-induced inflammation, insulin resistance, and alterations in tissue metabolism (Cnop et al., 2011; Gregor and Hotamisligil, 2011; Flamment et al., 2012). The activation of IKKβ/NF-κB and JNK by the IRE-1 arm of the UPR is one of these mechanisms (Gregor and Hotamisligil, 2011).

At the molecular level, one important mechanism by which IKKβ and JNK attenuate insulin signaling is the phosphorylation of IRS proteins on inhibitory serine phosphorylation sites (Figure 3). The mechanism by which IRS phosphorylation regulates insulin signaling is complex and described in details in recent reviews (Gual et al., 2005; Boura-Halfon and Zick, 2009; Tanti and Jager, 2009; Copps and White, 2012). In physiological condition, insulin induces a time-controlled phosphorylation of both positive and inhibitory serine sites in IRS1 and IRS2 in order to ensure the fine tuning of IRS tyrosine phosphorylation that is necessary for the propagation of insulin action (Tanti and Jager, 2009; Copps and White, 2012). Since our pioneer study (Tanti et al., 1994), it is now admitted that during obesity, activation of inflammatory, and stress kinases such as JNK and IKK is responsible for an uncontrolled phosphorylation of IRS on inhibitory serine sites resulting in a decrease in IRS tyrosine phosphorylation and a desensitization of insulin signaling (Boura-Halfon and Zick, 2009; Tanti and Jager, 2009). It is noteworthy that JNK seems more involved in the direct IRS serine phosphorylation than IKKβ (Tanti and Jager, 2009). IKKβ activation could promote IRS1 serine phosphorylation through activation of TSC1/TSC2/mTORC1/S6 Kinase-1 pathway leading to inhibitory IRS1 serine phosphorylation by S6K1 (Lee et al., 2008). Of note, activation of mTORC1 by IKKβ could be also involved in feed-forward mechanism induced by inflammatory cytokines to promote ER stress since over-activation of mTORC1 has been linked to the development of ER stress (Ozcan et al., 2008) (Figure 3). In addition, activation of the IKKβ/NFκB pathway increases the expression of PTP1B, a tyrosine phosphatase that dephosphorylates IRS1 (Zabolotny et al., 2008).

**Figure 4:**
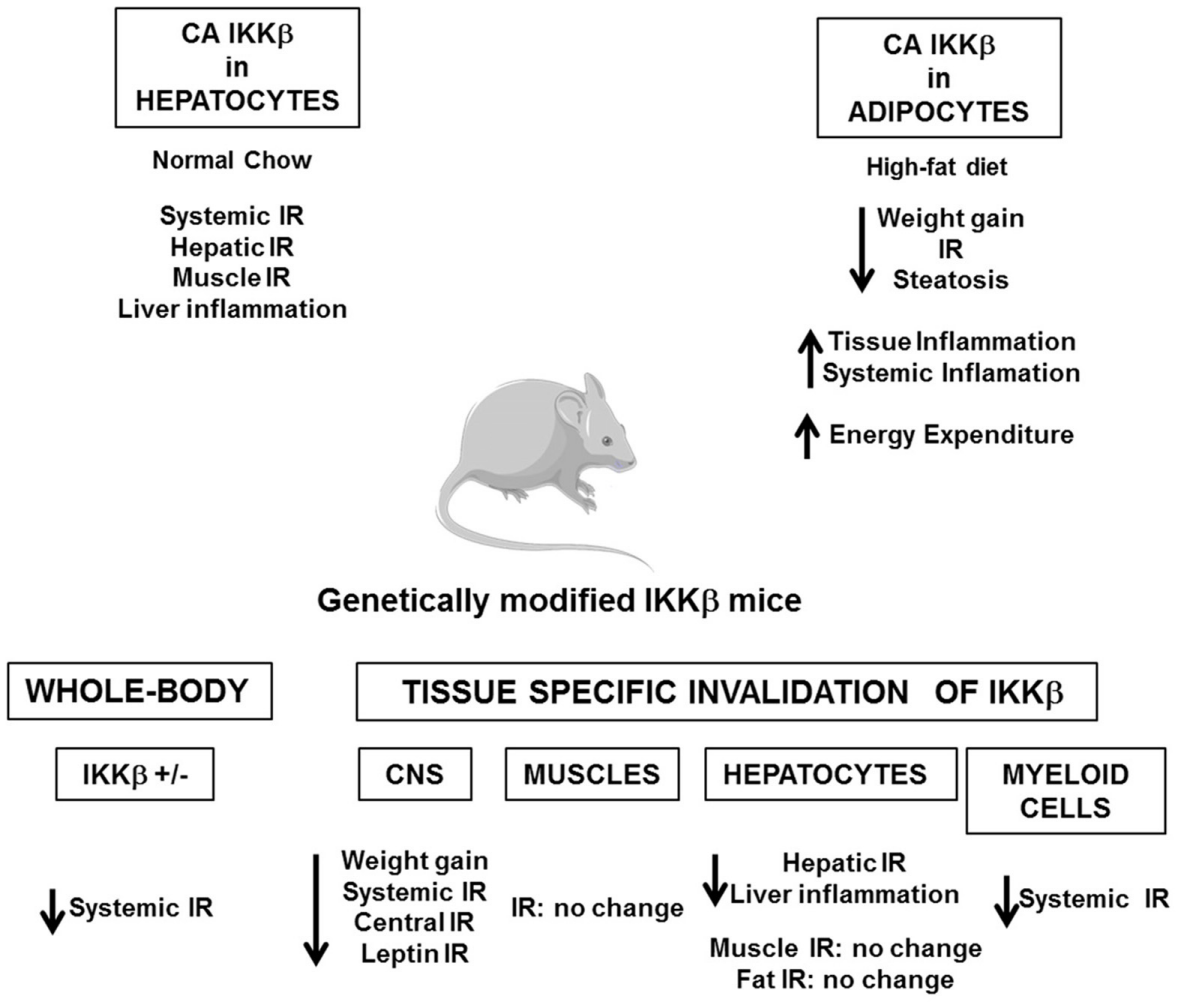
**Consequences of the genetic manipulation of IKKβ on the metabolic phenotype of mice.** Constitutive activation of IKKβ (CA IKKβ) in hepatocytes results in a deterioration of the insulin sensitivity of lean mice. At the opposite constitutive activation of IKKβ in adipocytes protects the mice against obesity and insulin resistance when fed a high-fat diet. Study of heterozygote IKKβ mice (IKKβ^+/-^) or of mice with a tissue specific inactivation of IKKβ in the central nervous system (CNS), hepatocytes, myeloid cells, or muscles demonstrates that activation of IKKβ/NF-κB pathway is a core mechanism that connects metabolic inflammation and insulin resistance in peripheral metabolic tissues, except muscles, and in the central nervous system.

**Figure 5:**
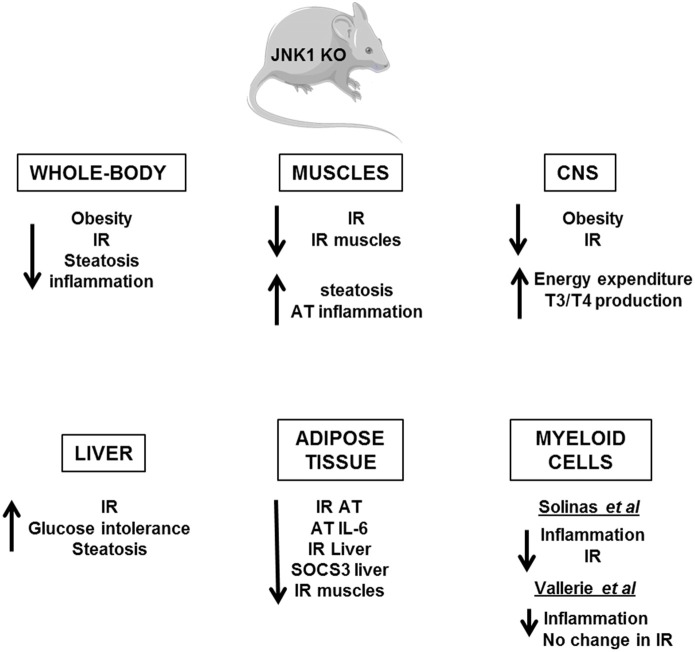
**Metabolic phenotype of whole-body JNK1 knockout mice and of conditional JNK1 knockout mice.** Whole-body invalidation of JNK1 protects mice against diet-induced obesity and insulin resistance. Tissue-specific invalidation of JNK1 reveals that the lack of JNK1 in adipose tissue, muscles, or CNS protects against the development of obesity and/or insulin resistance. The lack of JNK1 in myeloid cells decreases obesity-induced inflammation but the impact on insulin resistance development is controversial. At the opposite, the lack of JNK1 in the hepatocytes alters liver function suggesting a protective role of JNK1.

Several *in vivo* studies in mice have demonstrated the importance of the IKKβ/NF-κB and JNK pathways in the development of insulin resistance (Figures 4, 5). Heterozygous IKKβ and whole-body JNK1-deficient mice were partially protected against diet-induced insulin resistance (Yuan et al., 2001; Hirosumi et al., 2002). JNK2 could also play a role in insulin resistance but to a lesser extent (Tuncman et al., 2006). A cell-autonomous mechanism that involves the negative regulation of IRS1 function by serine phosphorylation was implicated in the regulation of insulin resistance induced by JNK1 and possibly by IKKβ during obesity (Hirosumi et al., 2002; Sabio et al., 2010b). IKKβ haplo-insufficiency or JNK1invalidation could also reduce the pro-inflammatory effect of high-fat diet. In this regard, mice lacking IKKβ or JNK1 in immune cells were partially protected against obesity-induced inflammation (Arkan et al., 2005; Solinas et al., 2007; Vallerie et al., 2008). However, if the protection against systemic insulin resistance was obvious for IKKβ deletion (Arkan et al., 2005), contradictory results were reported for JNK1 invalidation (Solinas et al., 2007; Sabio et al., 2008; Vallerie et al., 2008).

The picture that emerges is that activation of these pathways in non-hematopoietic cells also participates in tissues inflammation and local or systemic insulin resistance. However, the consequences of IKKβ and JNK activation on the development of insulin resistance could totally differ and depend on the site of action, the level of expression and the impact on adiposity. Indeed, the activation of IKKβ/NF-κB in hepatocytes-induced liver inflammation and insulin resistance (Arkan et al., 2005; Cai et al., 2005; Tamura et al., 2007; Wunderlich et al., 2008) and was associated with a reduced ability of insulin to suppress neoglucogenesis (Arkan et al., 2005) and with an increased production of VLDL leading to the development of hypertriglyceridemia (van Diepen et al., 2011). At the opposite of these findings, the development of inflammation induced by a moderate IKKβ activation in adipose tissue and before the onset of obesity has been shown to be protective against diet-induced insulin resistance by limiting adipose tissue expansion and by increasing energy expenditure (Jiao et al., 2012). Furthermore, in contrast to the deleterious effect of IKKβ, the activation of JNK pathway in liver seems to have a protective role since hepatocyte-specific invalidation of JNK1 led to the development of glucose intolerance, insulin resistance, and liver steatosis even in lean mice (Sabio et al., 2009). This finding was quite unexpected given that the liver of obese whole-body JNK1-deficient mice was more insulin sensitive with less inflammation. Thus, it is possible that the inhibition of JNK1 in hepatocytes *per se* is detrimental whereas the combined inhibition of JNK1 in hepatocytes and in other liver cells of the liver has a protective effect. Further, organ to organ communication, especially cross-talk between liver and adipose tissue, could be another explanation since JNK1 invalidation in adipose tissue ameliorated liver insulin sensitivity (Sabio et al., 2008; Zhang et al., 2011). One important mediator of this cross-talk could be the adipocytokines, especially IL-6, which altered liver insulin sensitivity through induction of SOCS3 (Sabio and Davis, 2010). The consequences of JNK1 or IKKβ activation in muscles are also totally different. While inactivation of IKKβ in cultured muscle cells markedly reduced cytokine-induced insulin resistance (Austin et al., 2008), the study of mice with conditional knockout of IKKβ in muscles argues against a major role of muscular IKKβ activation in obesity-associated insulin resistance (Rohl et al., 2004). Muscle-specific invalidation of JNK1 improved high-fat diet-induced muscles insulin resistance but also led to an enhanced liver steatosis and to a mild increase in inflammatory mediator expression in adipose tissue. The cross-talk between these different organs was mediated by an increase in circulating triglycerides owing to a reduction in lipoprotein lipase expression in muscles (Sabio et al., 2010b). Systemic insulin sensitivity was slightly improved in those mice suggesting that the improved insulin sensitivity in muscles was sufficient to overcome the enhanced liver steatosis and adipose tissue inflammation. However, it is possible that with age, a worsening in insulin sensitivity develops.

Several evidences suggest that activation of IKKβ and JNK pathways in the hypothalamus by over-nutrition contributes to energy imbalance and weight gain in addition to their role in the development of insulin resistance. At the molecular level, ER stress that develops in hypothalamus owing to an oversupply in nutriments activates the IKKβ/NF-κB pathway leading to local SOCS3 expression that interferes with both insulin and leptin signaling (Zhang et al., 2008). The mechanism by which JNK1 activation in the nervous system regulates body mass is different and complex. Two studies have revealed that invalidation of JNK1 in the nervous system markedly enhanced the production of thyroid hormones through the hypothalamic-pituitary-thyroid axis leading to an increase in energy expenditure and a protection against obesity (Belgardt et al., 2010; Sabio et al., 2010a).

These studies support the idea that the over-activation of IKKβ/NF-κB and JNK pathways is a core mechanism that connects metabolic inflammation and insulin resistance both in peripheral tissues and in the CNS. This central role highlights IKKβ and JNK as potential pharmacological targets against the development of insulin resistance. In this regard, Shoelson and colleagues have shown that high-doses of salicylate and its derivative salsalate were able to inhibit IKKβ activity and to improve insulin sensitivity in obese mice (Donath and Shoelson, 2011). Importantly, recent proof of concept studies and clinical trials in type 2 diabetic patients suggest that salsalate ameliorates glucose tolerance through better insulin sensitivity, and/or *via* an increased insulin secretion (Koska et al., 2009; Goldfine et al., 2010). The consequences of the pharmacological targeting of JNK have been studied only in mice. Competitive inhibitors of ATP directed against JNK and specific small substrate-competitive inhibitors of JNK displayed protective effects against diet-induced insulin resistance and/or weight gain (Bogoyevitch and Arthur, 2008; Cho et al., 2008; Yang and Trevillyan, 2008). These results suggest that IKKβ or JNK inhibitors could be interesting therapeutic agents against insulin resistance and type 2 diabetes. However, it should be kept in mind that IKKβ/NF-κB pathway is a central regulator of immunity and that JNK pathway regulates numerous physiologic processes. Chronic inhibition of these pathways might thus have side effects and favor the emergence of other pathologies.

### The extracellular signal-regulated kinases in obesity development and insulin resistance

The Extracellular signal-Regulated Kinases (ERK) 1/2 (also known as p44 and p42 MAP kinase) are activated by several growth factors but also by inflammatory cytokines. The activation of ERK1/2 requires the phosphorylation of both tyrosine and threonine residues located in a TEY sequence. This phosphorylation is mediated by the MAP kinase kinase (MAP2K) MEK. The activation of MEK also requires its phosphorylation by MAP kinase kinase kinase (MAP3K). Depending on the stimuli, different MAP3Ks are engaged to phosphorylate MEK. The activated ERK1/2 phosphorylates numerous substrates with serine or threonine residues close to a proline residue (Keshet and Seger, 2010). The activity of ERK1/2 is increased in adipose tissue, liver, and muscles of obese/diabetic patients or mice for review see Tanti and Jager (2009). Several cellular studies have shown that activation of ERK1/2 by diabetogenic factors-induced IRS1 serine phosphorylation. These phosphorylation events decrease the interaction between IRS1 and the PI3K or inhibit the association between IRS1 and the insulin receptor and would thus diminish the metabolic effects of insulin (Tanti and Jager, 2009). This mechanism has relevant implications in human pathology since basal ERK activity and IRS1 phosphorylation are abnormally increased in primary muscle cells from type 2 diabetic patients (Bouzakri et al., 2003). In addition, activation of the ERK pathway by the inflammatory cytokines, especially IL-1β, in adipocytes also induced a decrease in the transcription of IRS-1 mRNA, leading to a decrease in insulin signaling and glucose transport (Jager et al., 2007). ERK activation by inflammatory cytokines could also indirectly promote insulin resistance by the stimulation of adipocyte lipolysis and the release of free fatty acids (Souza et al., 2003).

The contribution of ERK pathway in the development of obesity and insulin resistance was first demonstrated by our study of ERK1-deficient mice (Bost et al., 2005). Those mice were protected against obesity when fed a high-fat diet, because of a decrease in adipogenesis and an increase in postprandial energy expenditure. The lack of obesity was associated with a better glucose and insulin tolerance compared to wild-type mice (Bost et al., 2005). Conversely, over-activation of the ERK pathway owing to deletion of the signaling adapter p62 resulted in the development of mature-onset obesity and insulin resistance with reduced energy expenditure and increased adipogenesis (Rodriguez et al., 2006). This phenotype was probably due to the over-activation of ERK1 rather than ERK2 since deletion of ERK1 in the p62^−/−^ genetic background reversed the phenotype (Lee et al., 2010). Those studies clearly highlighted the role of ERK1 in the development of obesity but did not allow concluding whether ERK1 could modulate the insulin sensitivity independently of its effect on body weight. However, we have recently shown that invalidation of ERK1 protected obese *ob/ob* mice against insulin resistance and adipose tissue inflammation without any changes in obesity (Jager et al., 2011). Another study also suggested that inhibition of ERK had beneficial effects on insulin resistance independently of an effect on body weight gain (Emanuelli et al., 2008a).

The pharmacological targeting of ERK1/2 against insulin resistance could have a series of drawbacks since they are involved in numerous biological processes. A possible alternative choice would be to target proteins which control ERK activity, specifically in response to inflammatory stresses which develop during obesity. In this regard, interesting candidates could be specific MAP3 kinases that are important in innate immune receptor signaling to MAP kinase activation (Symons et al., 2006).

Among the different MAP3K that control ERK activity, our team recently identified the kinase Tpl2 (Tumor progression locus, MAP3K8) as a potential new player in adipose tissue dysfunction and inflammation (Jager et al., 2010). In immune cells, Tpl2 plays an important role in the production of inflammatory cytokines, especially TNFα (Dumitru et al., 2000), and it is also involved in cytokines signaling downstream of innate immunoreceptors (Gantke et al., 2011). The importance of Tpl2 in inflammation has been demonstrated by the resistance to endotoxin shock and the lack of TNF-α production by macrophages of the Tpl2-deficient mice (Dumitru et al., 2000). Further, Tpl2 is a critical regulator of pancreatic, lung, and bowel inflammation in mice (Gantke et al., 2011). In non-stimulated cells, Tpl2 binds in its inactive form to p105NF-κB and is activated by different inflammatory stimuli through phosphorylation and degradation of p105NF-κB induced by IKKβ (Gantke et al., 2011). Among them, LPS, TNF-α, IL-1β, and CD40 are involved in obesity-induced inflammation and insulin resistance (Poggi et al., 2009; Tanti and Jager, 2009) suggesting a potential role for Tpl2 in this pathology. Recently, we found Tpl2 up-regulated in adipose tissue of obese mice and patients. Tpl2 was involved in the lipolytic effect the inflammatory cytokines and in the serine phosphorylation of IRS1 in adipocytes (Jager et al., 2010). Based on these findings, one can hypothesize that the targeting of Tpl2 could have beneficial effects in the context of obesity by reducing the production of inflammatory cytokines by adipose tissue immune cells and by blocking their deleterious effects in adipocytes. In agreement, one recent study has shown that Tpl2 inactivation protected the mice against insulin resistance with a reduction in liver and adipose tissue inflammation (Perfield et al., 2011). However, another study failed to confirm this finding (Lancaster et al., 2012). Thus, further studies are needed to conclude whether the targeting of Tpl2 could improve the complications of obesity.

## Conclusion

The discovery that metabolic diseases are associated with a low-grade inflammatory state has opened a new area of research to understand how inflammation develops and how it impact on metabolic pathways. It appears that a cross-talk between immune cells and metabolic cells plays a central role in the disturbance of metabolic homeostasis. High levels of dietary saturated fatty acids or of their metabolites can be detected by immune sensors such as TLR or inflammasome leading to the synthesis of inflammatory cytokines in different metabolic tissues. Dietary fat can also modify the intestinal microbiota that produces different inflammatory molecules leading to an inappropriate immune reaction. The inflammatory cytokines, saturated fatty acids, and LPS activate a network of signaling pathways that impinges on insulin signaling leading to alterations in metabolic cell functions. Hence, the importance of immune sensors and of different kinases suggests that new strategies targeting these proteins could be conceived to improve the metabolic complications of obesity.

### Conflict of interest statement

The authors declare that the research was conducted in the absence of any commercial or financial relationships that could be construed as a potential conflict of interest.
